# Phytoremediation of Metals and Nutrients by Aquatic Macrophytes in an Urban Semiarid River (Mossoró, Brazil)

**DOI:** 10.1021/acsomega.5c11892

**Published:** 2026-10-22

**Authors:** Adler L. S. da Silva, Talita D. Pedrosa, Rafael R. de Melo, Solange A. G. Dombroski, Tereza A. L. C. G. Rocha, Phâmella K. P. Farias, Daniela da C. L. Coelho, Stefeson B. de Melo, Silvanete S. da Silva, Francisca G. dos S. Bezerra, Rafael O. Batista

**Affiliations:** † Department of Engineering and Environmental Sciences, Universidade Federal Rural do Semi-Árido, Avenida Francisco Mota, 572, Costa e Silva, campus Mossoró, Mossoró 59625-900, Brazil; ‡ Department of Agronomic and Forestry Sciences, Universidade Federal Rural do Semi-Árido, Avenida, Francisco Mota, 572, Costa e Silva, campus Mossoró, Mossoró 59625-900, Brazil; § Department of Teaching, Research, Graduate Studies, Innovation, and Extension, Instituto Federal do Pará, Rodovia PA 275, s/n, Bairro União, Campus Parauapebas, Parauapebas 68515-000, Brazil; ∥ Academic Unit of Belo Jardim, Universidade Federal Rural de Pernambuco, Rodovia, PE 166, 100, Belo Jardim 55150-000, Brazil

## Abstract

Urban expansion and riverbank anthropogenic pressures have intensified the elemental loading of urban rivers, a problem that is exacerbated in semiarid regions where low flows, long residence times, and evaporation favor solute concentration and changes in aqueous speciation. Here, we quantified nutrients and trace metals and assessed macrophyte bioaccumulation and translocation in the urban reach of the Apodi-Mossoró River. Water and plant tissues of *Nymphaea alba* and *Eichhornia crassipes* were collected in eight campaigns across six sites during dry and rainy seasons. The elemental suite comprised N, P, Na, K, Ca, Mg, Cu, Zn, Fe, Mn, Cd, Cr, Ni, and Pb. Descriptive statistics and multivariate analyses evidenced element-specific patterns driven by spatial gradients and climatic seasonality that influence metal mobility and bioavailability. Phytoremediation performance was species dependent: *Nymphaea alba* displayed higher bioaccumulation and translocation for Pb (16,669.64) and Cu (9.32), whereas *Eichhornia crassipes* showed greater accumulation and translocation for Cr (11,291.96) and Ni (23.23). Overall, the contrasting uptake and internal partitioning of trace metals, together with seasonal variability in nutrient concentrations, support the use of *Nymphaea alba* and *Eichhornia crassipes* as complementary biomonitors and as candidate species for phytoremediation of metal and nutrient contamination in urban semiarid rivers.

## Graphical abstract

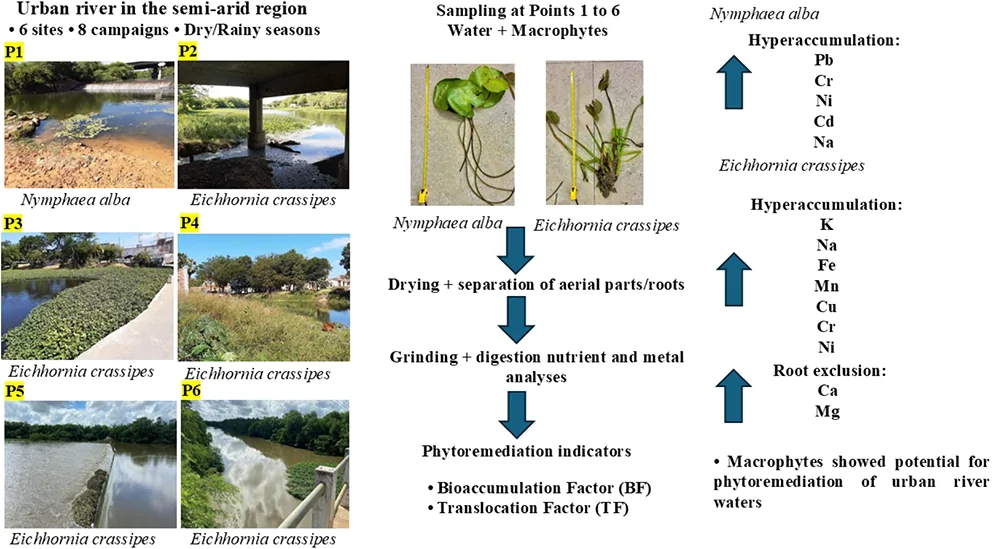

## 1 Introduction

Rivers are dynamic ecosystems that support high biodiversity and play essential roles in global biogeochemical cycles, nutrient transport, and ecological balance.^1^ However, the natural functioning of these systems is increasingly influenced by anthropogenic activities. In semiarid regions, such as northeastern Brazil, seasonal rainfall patterns significantly affect river hydrology, altering flow regimes and nutrient dynamics.^2^ The Apodi-Mossoró River, which crosses the municipality of Mossoró, exemplifies this variability, as its discharge fluctuates between dry and rainy seasons and is further modified by the presence of dams.^3^

Hydrological alterations, such as damming, can transform natural lotic systems into lentic or hybrid environments, leading to changes in sedimentation rates, oxygen dynamics, and contaminant retention.^4^ These alterations, coupled with rapid industrial and agricultural expansion, have accelerated the deterioration of surface water quality by increasing the fluxes of dissolved and particulate chemical species to riverine systems. As a result, rivers act as sinks and reactive conduits for complex mixtures of pollutants, including labile organic matter and trace metals delivered via domestic effluents, agricultural runoff, and atmospheric deposition. At the elemental and molecular levels, these inputs modify ionic composition and strength, introduce organic ligands that complex metal ions, and drive sorption–desorption and redox transformations, thereby controlling metal speciation, mobility, and bioavailability in the aquatic environment.^5,6^

Trace metals are of particular concern because they are not degraded, can remain in the aquatic system through changes in chemical speciation and partitioning, and may persist in bioavailable forms. Their toxicity to aquatic organisms is strongly controlled by elemental and molecular factors, including oxidation state, aqueous complexation with inorganic ions and natural organic ligands, and adsorption onto reactive mineral, particulate, and biological surfaces, which together regulate mobility, uptake, and bioaccumulation.^7^ Although some metals occur naturally, their increased input from human sources elevates concentrations to harmful levels.^8^ Toxic metals and metalloids such as As, Cd, Cr, Hg, and Pb can bioaccumulate in food chains, posing severe risks to aquatic life and human health through damage to the kidneys, liver, and nervous system, as well as carcinogenic potential.^9,10^ Metal toxicity often results from transformations into ionic or organometallic fractions through biomethylation processes, which enhance their mobility and bioavailability in water.^11,12^

Given these impacts, water pollution has become one of the most pressing environmental challenges of the twenty-first century. The need for sustainable and low-cost remediation strategies has driven attention toward biological approaches, particularly those that use plants and microorganisms to remove contaminants from water and sediments.^13^ Among these, phytoremediation stands out as an environmentally viable and effective technique that harnesses the natural capacity of plants to absorb, accumulate, and mitigate contaminants.^14,15^

Within this context, certain macrophyte species have demonstrated remarkable potential for removing excess nutrients and heavy metals from contaminated waters. *Eichhornia crassipes* (water hyacinth), native to the Amazon basin, is recognized for its rapid growth, extensive fibrous root system, and high capacity to accumulate elements above physiological needs.^16–18^ Its long, dense roots increase the surface area for adsorption and uptake, enhancing the removal of metals such as Fe, Zn, and Pb from contaminated water bodies.^19^ Similarly, *Nymphaea alba* (white water lily) is a rooted macrophyte with perennial rhizomes, capable of reflecting environmental changes in trophic status and serving as a bioindicator in aquatic ecosystems.^20,21^

The efficiency of a plant species for phytoremediation depends primarily on two physiological processes: bioaccumulation the uptake and incorporation of elements into plant tissues and translocation the transfer of these elements from roots to aerial parts.^22,23^ According to their capacity to accumulate contaminants, plants can be classified as accumulators, indicators, or excluders, depending on the concentration ratios between plant tissues and the surrounding environment.^24^

Despite the growing number of studies on phytoremediation, research focusing on macrophyte metal dynamics in semiarid rivers remains limited, especially in South America. Semiarid environments present unique hydrological and geochemical conditions that influence contaminant mobility and plant uptake, making such ecosystems ideal natural laboratories for evaluating phytoremediation potential.

Therefore, the present study aimed to quantify the spatiotemporal bioaccumulation and internal translocation of metals in *Eichhornia crassipes* and *Nymphaea alba* along the urban stretch of the Apodi-Mossoró River. Our hypothesis was that these macrophytes exhibit distinct capacities for metal absorption and intraplant partitioning under semiarid conditions. Furthermore, we expected *Eichhornia crassipes* to exhibit greater efficiency in metal accumulation.

## 2 Materials and Methods

### 2.1 Location and Characterization of the Study Area

Located in the Middle and Upper West region of the state of Rio Grande do Norte, the Apodi-Mossoró River basin extends 210 km, covering an area of 14,276 km^2^, equivalent to 26.8% of the state’s territory. The source of the Apodi-Mossoró River is located on the border with the state of Paraíba, in the city of Luís Gomes-RN, and its mouth is located on the Atlantic coast, between the cities of Grossos-RN and Areia Branca-RN, Brazil.^25^ The Apodi-Mossoró River crosses the city of Mossoró, passing through several neighborhoods. Figure 1 shows the urban section of the Apodi-Mossoró River, highlighting the six points where water and macrophyte samples were collected.

**Figure 1:**
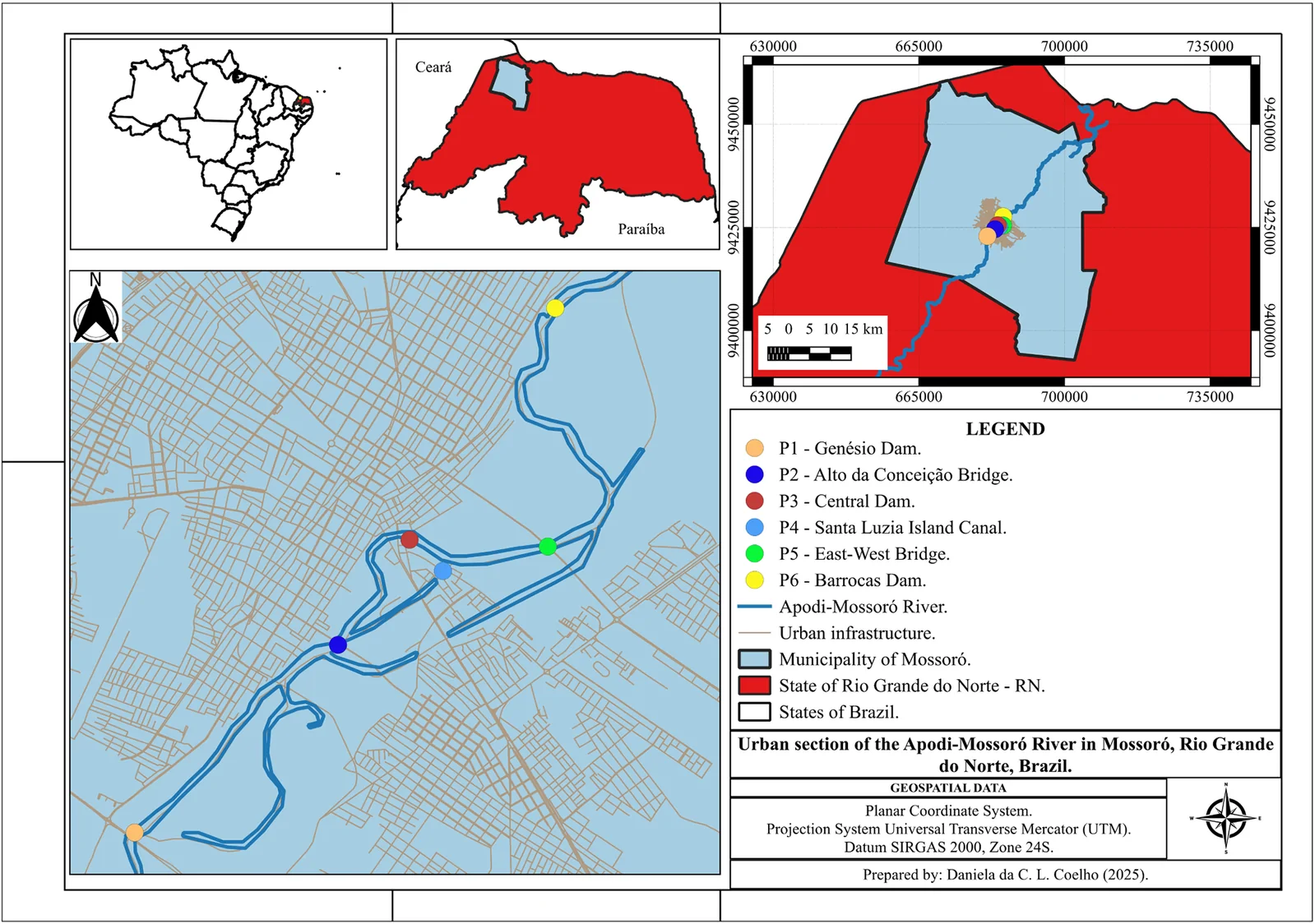
Image of the urban section of the Apodi-Mossoró River in Mossoró, Rio Grande do Norte, Brazil, highlighting the six collection points for water and macrophyte samples.

The evaluated region comprises soils classified by the Empresa Brasileira de Pesquisa Agropecuária (EMBRAPA) as Latossolos Vermelho-Amarelos, Argissolos Vermelho-Amarelos, Neossolos Quartzarênicos, Cambissolos Háplicos, and Neossolo Litólico. These soils correspond, respectively, to the World Reference Base for Soil Resources (WRB) soil groups Ferralsols, Acrisols, Arenosols, Cambisols, and Leptosols.^26,27^

Eight campaigns were carried out to collect water and macrophyte samples (*Eichhornia crassipes* and *Nymphaea alba*) between July 2022 and April 2023, on the following dates: 07/26/22, 08/23/22, 09/20/22, 10/25/22, 12/03/22, 01/26/23, 02/26/23, and 04/10/23, at six points along the urban stretch of the Apodi-Mossoró River in Mossoró (Table 1).

**Table 1:** Collection Points, Description of Sampling Locations, and Geographic Coordinates

Collection Points	Description of sampling locations	Geographical coordinates of collection points	Plane coordinates
P1	Genésio Dam	5°13′9.58″S/37°21′45.53″W	9422855.54 m S/681470.42 m E/24S
P2	Alto da Conceição Bridge	5°12′13.8″S/37°20′46.1″W	9424564.20 m S/683305.06 m E/24S
P3	Centro Dam	5°11′42.60″S/37°20′25.21″W	9425520.92 m S/683950.91 m E/24S
P4	Santa Luzia Island Canal	5°11′51.8″S/37°20′15.4″W	9425237.52 m S/684252.28 m E/24S
P5	East–West Bridge	5°11′44.5″S/37°19′44.7″W	9425459.27 m S/685198.32 m E/24S
P6	Barrocas Dam	5°10′33.83″S/37°19′42.68″W	9427629.97 m S/685266.25 m E/24S

The selected collection points are distributed between the Genésio dam, located on the banks of the BR-304 highway, and the Barrocas neighborhood, covering approximately 10 km along the river. The choice of these collection sites was based on an on-site visit through a preliminary visual assessment, carried out before the initial collection of samples to compare anthropized areas with more conserved areas. *Eichhornia crassipes* macrophytes were found at all collection points except P1, where *Nymphaea alba* macrophytes predominated, as shown in the images in Figure 2.

**Figure 2:**
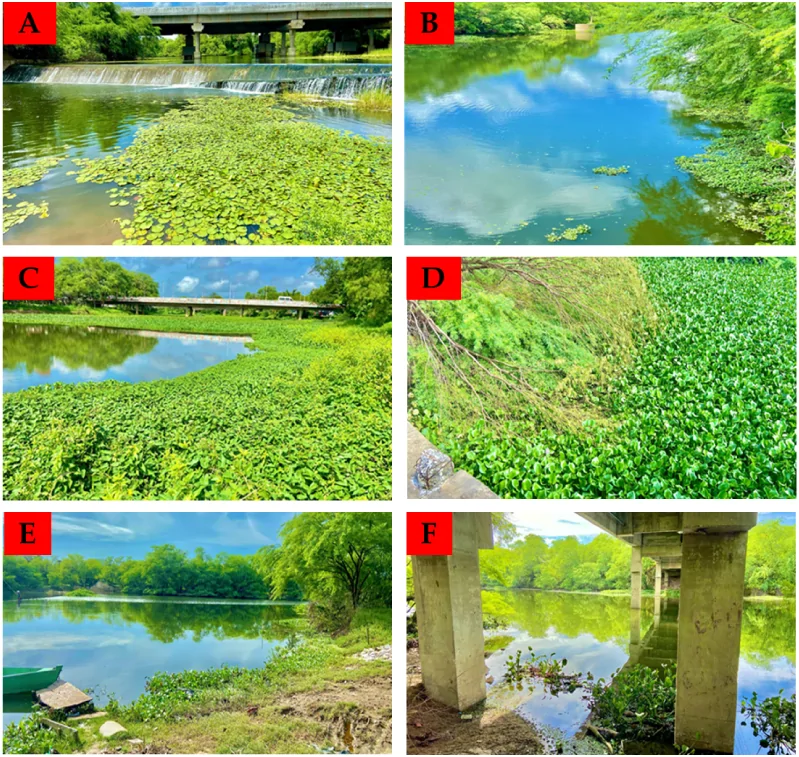
Images of the urban section of the Apodi-Mossoró River in Mossoró highlighting the Genésio Dam-Point 1 (A), Alto da Conceição Bridge-Point 2 (B), Centro Dam-Point 3 (C), Santa Luzia Island Canal-Point 4 (D), East–West Bridge-Point 5 (E), and Barrocas Dam-Point 6 (F).

P1: This sampling point is located at the first weir (Figure 2A), within the urban perimeter, in an area adjacent to the RN-304 highway, which has an estimated daily traffic of 6,000 vehicles, including a significant proportion of heavy-duty vehicles. The highway constitutes one of the main logistical corridors in Northeastern Brazil, playing a strategic role in the transportation of agricultural and industrial products, as well as passenger mobility and regional tourism. In addition, this site is subject to multiple anthropogenic uses, including recreational activities (bathing), vehicle washing, and continuous water abstraction for urban landscaping irrigation, all of which may directly influence water quality.

P2: This sampling point is located downstream of a major avenue in the municipality, in the Alto da Conceição neighborhood (Figure 2B). The area is characterized by intense anthropogenic interference and is used by animals and unhoused individuals for personal hygiene activities, clothes washing, often involving cleaning products, and other daily practices. Furthermore, the presence of stormwater drainage channels and illegal sewage discharges is observed, which may significantly compromise water quality.

P3: This sampling point is located at a weir (Figure 2C) situated in the central region of the city. The area exhibits a high degree of sedimentation, along with a significant presence of sewage discharges, stormwater drainage channels, solid waste, and associated byproducts. Low or negligible water movement is observed, particularly during the dry season, conferring eutrophic characteristics to the environment because of intense anthropogenic pressure.

P4: This sampling point is in the lower reach of the river, corresponding to the tripartite river zoning, in the Ilha de Santa Luzia neighborhood (Figure 2D). The area receives a high load of urban sewage and solid waste. Visually, the site exhibits a high degree of environmental contamination, a condition commonly associated with watercourses located in densely urbanized areas.

P5: This sampling point is located downstream of a bridge along an east–west arterial avenue (Figure 2E), characterized by intense vehicular traffic. This river stretch presents greater water depth compared to the other sampling points. In the surrounding area, the presence of riparian vegetation is observed, and significant changes in water characteristics were recorded throughout the monitoring period. Additionally, drainage from an adjacent establishment is discharged into the river, representing a potential source of water quality impairment.

P6: The last sampling point is located at a weir in the Barrocas neighborhood (Figure 2F). This river stretch presents a high-water volume and is used by local residents for fishing activities and animal bathing. Sewage discharges from residences located along the riverbanks were identified, as well as the continuous improper disposal of solid waste, factors that may compromise the environmental quality of the water body.

### 2.2 Climate Aspects

Mossoró is a municipality located in the western region of Rio Grande do Norte, with a hot and dry climate (BSh), with rainfall concentrated in a few months of the year and long periods of drought in the remaining months, mainly from September to December.^28^ The municipality has an average altitude of 55.0 m, average annual rainfall of 794 mm, and average air temperature of 26.5 °C.^29^

During the experimental period, eight campaigns were carried out to collect water and macrophyte samples at six points along the urban stretch of the Apodi-Mossoró River in Mossoró, with representation of these campaigns in the dry and rainy seasons. During this period, the climatological variables of rainfall and solar radiation were monitored to assist in explaining the fluctuations in the river water attributes. Rainfall and solar radiation data were obtained from an automatic weather station installed on the East Campus of Universidade Federal Rural do Semi-Árido (UFERSA) Mossoró-RN. Figure 3 shows the monthly accumulated rainfall and accumulated solar radiation on the day of each collection.

**Figure 3:**
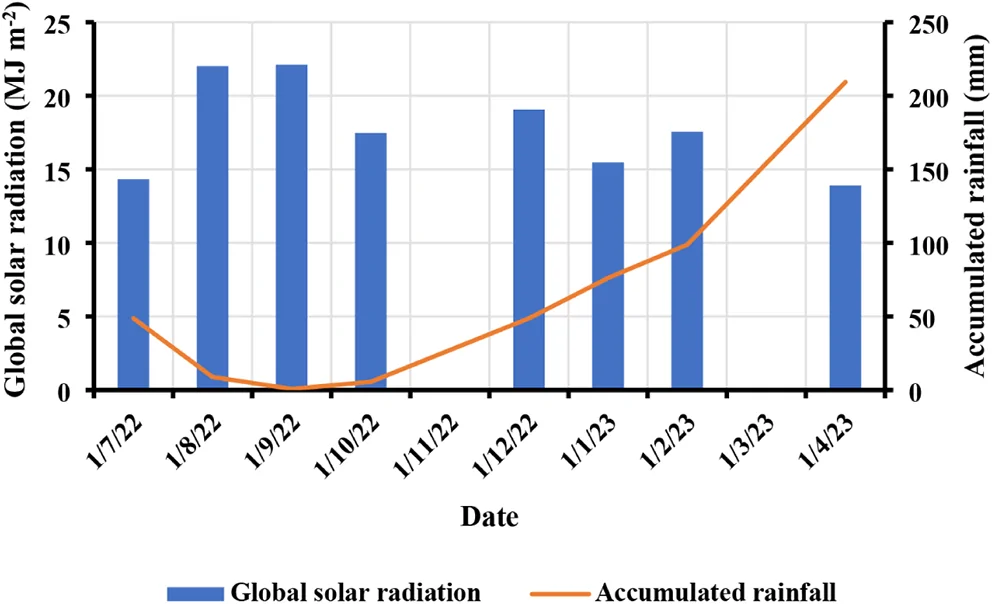
Monthly accumulated rainfall and global solar radiation on the day each water and macrophyte sample was collected.

### 2.3 Collection and Analysis of Water Samples

During the experimental period, eight campaigns were carried out to collect and analyze chemical attributes in the water at six sampling points along the urban stretch of the Apodi-Mossoró River in Mossoró (Table 2).

**Table 2:** Mean Values and Standard Deviation of Chemical Attributes of Water Samples Collected (N = 8)^a^

	Mean and standard deviation					
Chemical elements	P1 (*Nymphaea alba*)	P2 (*Eichhornia crassipes*)	P3 (*Eichhornia crassipes*)	P4 (*Eichhornia crassipes*)	P5 (*Eichhornia crassipes*)	P6 (*Eichhornia crassipes*)
TKN (mg L^–1^)	1.75 ± 0.86	2.74 ± 1.94	4.20 ± 1.85	8.19 ± 4.99	11.84 ± 9.18	8.08 ± 3.06
Pt (mg L^–1^)	0.04 ± 0.05	0.03 ± 0.01	0.05 ± 0.05	0.13 ± 0.10	0.098 ± 0.08	0.23 ± 0.32
K^+^ (mmolc L^–1^)	0.19 ±0.08	0.19 ± 0.06	0.21 ± 0.06	0.19 ± 0.06	0.22 ± 0.08	0.15 ± 0.06
Ca^2+^ (mmolc L^–1^)	3.29 ± 2.11	4.03 ± 1.66	3.43 ± 1.94	4.36 ± 1.61	4.01 ± 1.29	4.60 ± 1.63
Mg^2+^ (mmolc L^–1^)	4.58 ± 2.84	2.20 ± 3.19	2.62 ± 1.67	3.76 ± 1.76	2.67 ± 3.91	3.79 ± 1.17
Na^+^ (mmolc L^–1^)	6.46 ± 2.82	6.12 ± 2.76	6.90 ± 3.44	7.40 ± 3.85	6.22 ± 3.44	7.60 ± 3.36
Fe (mg L^–1^)	17.7 ± 6.26	12.33 ± 21.32	29.40 ± 10.39	38.55 ± 23.09	0.60 ± 14.73	6.68 ± 15.64
Mn (mg L^–1^)	6.69 ± 7.31	3.80 ± 5.50	3.79 ± 3.68	10.35 ± 10.76	2.85 ± 3.44	17.02 ± 24.95
Cu (mg L^–1^)	0.45 ± 0.01	0.50 ± 0.01	0.15 ± 0.05	0.001 ± 0.00	0.001 ± 0.00	0.001 ± 0.00
Zn (mg L^–1^)	6.00 ± 13.94	1.50 ± 1.31	0.82 ± 1.25	1.26 ± 1.35	1.69 ± 1.50	2.33 ± 2.80
Cr (mg L^–1^)	0.001 ± 0.00	0.15 ± 0.01	0.001 ± 0.00	0.001 ± 0.00	0.001 ± 0.00	0.001 ± 0.00
Pb (mg L^–1^)	0.001 ± 0.00	0.37 ± 0.01	1.50 ± 0.01	1.00 ± 0.01	3.00 ± 0.01	2.75 ± 0.53
Cd (mg L^–1^)	0.001 ± 0.00	0.001 ± 0.00	0.01 ± 0.00	0.05 ± 0.01	0.01 ± 0.00	0.10 ± 0.10
Ni (mg L^–1^)	0.001 ± 0.00	0.001 ± 0.00	0.001 ± 0.00	0.001 ± 0.00	0.001 ± 0.00	0.10 ± 0.00

a P1—Genésio Dam; P2—Alto da Conceição Bridge; P3—Centro Dam; P4—Santa Luzia Island Canal; P5—East–West Bridge; and P6—Barrocas Dam.

For the collection and analysis of water samples from the urban section of the Apodi-Mossoró River, the recommendations of Standard Methods for the Examination of Water and Wastewater^30^ and EMBRAPA^31^ were used.

Water samples were collected *in situ* using 1 L polyethylene bottles, which were immediately preserved after collection in insulated boxes with ice at 4 °C until arrival at the laboratories of the UFERSA, east campus Mossoró-RN, for chemical analysis. The river water quality analyses were performed at the Soil, Water, and Plant Laboratory (LASAP) and the Sanitation Laboratory (LASAN).

The chemical attributes analyzed were as follows: total Kjeldahl nitrogen (TKN), which refers to the combination of ammonia and organic nitrogen, using the Kjeldahl digestion method; total phosphorus (Pt), obtained by the ascorbic acid method with preliminary digestion using the persulfate method; available potassium (K^+^) and available sodium (Na^+^), determined by flame photometry; available calcium (Ca^2+^) and available magnesium (Mg^2+^), quantified by complexometry with EDTA, using eriochrome black T as an indicator; and the heavy metals iron (Fe), manganese (Mn), copper (Cu), zinc (Zn), chromium (Cr), lead (Pb), cadmium (Cd), and nickel (Ni) were preserved with HNO_3_^–^ and refrigerated at 4 °C and determined by atomic absorption spectrophotometry. The water samples were not subjected to digestion, but were instead filtered to remove suspended particles, with subsequent readings.

### 2.4 Collection and Analysis of Macrophyte Samples

In parallel with water sampling, the predominant macrophyte species was collected at each of the six sampling sites of the surface water body during the eight sampling campaigns. The macrophyte samples were collected and stored in polyethylene bags, transported in an insulated box with ice, and preserved at 4 °C until arrival at the laboratories for subsequent chemical analysis. All analytical procedures were conducted at LASAP/UFERSA, LASAN/UFERSA, and the Laboratory for Analysis of Soil, Water, and Plants of the Semi-Arid Region (LASAPSA/UFERSA).

At LASAN/UFERSA, macrophyte samples were washed with running fresh water to remove adhering residues and minimize potential interference with analytical results. The samples were then weighed using a digital electronic balance to determine their fresh mass. Subsequently, the plant tissues were air-dried at room temperature to remove excess moisture remaining from the initial washing. The mean percentage of water was estimated using the means values for fresh and dry mass of macrophytes (Table 3).

**Table 3:** Mean Values and Standard Deviation of Fresh and Dry Masses of Macrophyte Samples and Their Respective Mean Percentage of Water (N = 8)^a^

Points	Fresh weight (g)	Dry weight (g)	Mean percentage of water (%)
P1 (*Nymphaea alba*)	114.29 ± 52.07	16.00 ± 6.11	86
P2 (*Eichhornia crassipes*)	244.65 ± 125.47	18.15 ± 11.05	93
P3 (*Eichhornia crassipes*)	148.79 ± 43.19	11.84 ± 4.46	92
P4 (*Eichhornia crassipes*)	317.80 ± 107.99	24.55 ± 12.14	92
P5 (*Eichhornia crassipes*)	358.62 ± 189.45	25.67 ± 14.70	93
P6 (*Eichhornia crassipes*)	298.24 ± 147.70	23.40 ± 11.42	92

a P1—Genésio Dam; P2—Alto da Conceição Bridge; P3—Centro Dam; P4—Santa Luzia Island Canal; P5—East–West Bridge; and P6—Barrocas Dam.

These samples were then washed six times with distilled water, according to the guidelines of,^32^ and subsequently immersed in a 20 mM Na2-EDTA solution for 20 min to remove heavy metals adhered to the root surfaces.^33^ The aerial part was then separated from the root system, and these separate samples were placed in a forced-air circulation oven at 65 °C for 72 h until the mass was completely stabilized. After this, the dry mass of the macrophytes was determined using the gravimetric method with a digital electronic scale.^32^

After drying, the macrophyte samples were ground in Wiley mills and stored in polyethylene containers with lids to minimize moisture ingress. The samples were then digested in a microwave oven using concentrated nitric acid.^34^

The attributes analyzed in the plant tissue digested in the microwave were: phosphorus (P), sodium (Na), potassium (K), calcium (Ca), magnesium (Mg), copper (Cu), zinc (Zn), iron (Fe), manganese (Mn), cadmium (Cd), chromium (Cr), nickel (Ni), and lead (Pb). This technique involves digesting a 0.5 g sample of crushed plant tissue in the presence of nitric acid, with a volume of 5 mL of 65% HNO_3_ diluted with up to 25 mL of water. This mixture was placed in Teflon tubes and subjected to a temperature ranging from 170 to 180 °C for a period of 10 min.^31^

Specifically for nitrogen (N), digestion was performed in a digestion block in the presence of sulfuric acid. To determine N, sulfuric digestion (Kjeldahl) was performed using 0.1 g of a previously crushed sample and adding 1 g of the salt mixture (K_2_SO_4_ + CuSO_4_ in a 10:1 ratio), 3 mL of 98% H_2_SO_4_, and 1 mL of 30% H_2_O_2_. It was then slowly heated to 350 °C and subsequently titrated following the Kjeldahl principle. In this method, the NH_4_^+^ product of digestion is distilled in a strongly alkaline medium, and the condensate is collected in an Erlenmeyer flask containing boric acid solution, which is then titrated with acid.^31^

The plant tissue extract samples were subjected to reading in a UV–vis spectrophotometer at 660 nm to obtain P; to a flame photometer to determine K and Na; to titrimetry to quantify Ca and Mg; and to atomic absorption spectrophotometry to measure the levels of heavy metals Cu, Zn, Fe, Mn, Cd, Cr, Ni, and Pb. The determination of chemical elements in macrophyte plant tissue followed the recommendations of EMBRAPA.^31^ Tables 4 and 5 present the concentrations of chemical elements in the aerial and root tissues of macrophytes, respectively.

**Table 4:** Concentrations of Chemical Elements in the Aerial Plant Tissues of Macrophytes (N = 8)^a^

	Mean and standard deviation					
Chemical Elements	P1 (*Nymphaea alba*)	P2 (*Eichhornia crassipes*)	P3 (*Eichhornia crassipes*)	P4 **(** *Eichhornia crassipes*)	P5 **(** *Eichhornia crassipes*)	P6 (*Eichhornia crassipes*)
N (mg kg^–1^)	15.93 ± 6.07	18.20 ± 4.99	25.98 ± 8.59	25.25 ± 7.61	15.91 ± 7.04	14.59 ± 3.14
P (mg kg^–1^)	0.08 ± 0.02	0.16 ± 0.04	0.15 ± 0.05	0.16 ± 0.03	0.15 ± 0.09	0.13 ± 0.03
K^+^ (mg kg^–1^)	2.16 ±0.72	4.10 ± 1.47	3.87 ± 1.52	5.16 ± 0.75	5.31 ± 1.70	3.79 ± 1.18
Ca^2+^ (mg kg^–1^)	0.05 ± 0.04	0.04 ± 0.03	0.01 ± 0.01	0.02 ± 0.01	0.04 ± 0.04	0.04 ± 0.04
Mg^2+^ (mg kg^–1^)	0.04 ± 0.01	0.05 ± 0.02	0.04 ± 0.03	0.03 ± 0.01	0.07 ± 0.04	0.09 ± 0.08
Na^+^ (mg kg^–1^)	2387.52 ± 540.65	2774.33 ± 520.95	2080.74 ± 838.02	1733.97 ± 737.28	3775.52 ± 1592.02	2267.48 ± 688.30
Fe (mg kg^–1^)	1076.17 ± 1210.83	906.98 ± 1007.55	1692.80 ± 1468.41	1147.66 ± 2299.49	788.17 ± 1131.83	646.14 ± 907.16
Mn (mg kg^–1^)	471.99 ± 404.00	379.32 ± 213.48	582.53 ± 718.63	407.78 ± 847.43	564.69 ± 474.07	494.43 ± 404.78
Cu (mg kg^–1^)	0.82 ± 0.24	0.64 ± 0.87	5.92 ± 8.32	4.89 ± 9.06	0.25 ± 0.79	1.57 ± 1.16
Zn (mg kg^–1^)	11.69 ± 2.82	8.75 ± 5.23	26.56 ± 9.39	13.84 ± 11.71	14.61 ± 6.41	17.79 ± 6.69
Cr (mg kg^–1^)	2.99 ± 3.41	1.61 ± 1.70	9.27 ±10.27	0.56 ± 1.09	1.65 ± 4.67	5.14 ± 5.44
Pb (mg kg^–1^)	7.36 ± 4.91	8.21 ± 3.05	13.00 ± 10.00	9.14 ± 1.85	5.74 ± 5.06	13.25 ± 6.15
Cd (mg kg^–1^)	0.74 ± 0.72	0.46 ± 0.51	0.34 ± 0.57	0.25 ± 0.19	0.09 ± 0.16	0.94 ± 1.12
Ni (mg kg^–1^)	3.02 ± 2.45	1.99 ± 1.26	4.91 ± 5.76	1.85 ± 1.06	2.16 ± 2.22	6.23 ± 5.24

a P1—Genésio Dam; P2—Alto da Conceição Bridge; P3—Centro Dam; P4—Santa Luzia Island Canal; P5—East–West Bridge; and P6—Barrocas Dam.

**Table 5:** Concentrations of Chemical Elements in the Root System Tissues of Macrophytes (N = 8)^a^

	Mean and standard deviation					
Chemical elements	P1 (*Nymphaea alba*)	P2 (*Eichhornia crassipes*)	P3 (*Eichhornia crassipes*)	P4 (*Eichhornia crassipes*)	P5 (*Eichhornia crassipes*)	P6 (*Eichhornia crassipes*)
N (mg kg^–1^)	12.53 ± 5.17	13.94 ± 2.79	16.43 ± 1.59	13.85 ± 3.35	14.31 ± 3.57	15.21 ± 3.94
P (mg kg^–1^)	0.08 ± 0.02	0.13 ± 0.04	0.14 ± 0.02	0.14 ± 0.04	0.12 ± 0.08	0.09 ± 0.03
K^+^ (mg kg^–1^)	3.87 ± 2.77	3.44 ± 1.37	4.85 ± 0.80	4.55 ± 1.04	4.36 ± 1.73	3.14 ± 1.26
Ca^2+^ (mg kg^–1^)	0.05 ± 0.02	0.03 ± 0.01	0.01 ± 0.00	0.03 ± 0.01	0.06 ± 0.04	0.03 ± 0.01
Mg^2+^ (mg kg^–1^)	0.03 ± 0.01	0.05 ± 0.02	0.01 ± 0.01	0.07 ± 0.03	0.05 ± 0.02	0.06 ± 0.03
Na^+^ (mg kg^–1^)	3041.09 ± 1744.31	2600.93 ± 647.69	2480.89 ± 555.42	1582.23 ± 719.33	3086.10 ± 940.61	1580.56 ± 652.34
Fe (mg kg^–1^)	716.05 ± 476.71	710.06 ± 488.01	675.91 ± 1156.65	289.26 ± 120.52	479.92 ± 296.74	1197.12 ± 1727.52
Mn (mg kg^–1^)	311.79 ± 205.07	443.02 ± 489.65	144.89 ± 161.27	291.54 ± 323.54	476.70 ± 249.16	1534.62 ± 2381.47
Cu (mg kg^–1^)	0.47 ± 0.48	1.76 ± 1.29	4.36 ± 5.43	0.53 ± 0.35	0.56 ± 0.43	1.24 ± 1.28
Zn (mg kg^–1^)	8.32 ± 4.20	20.57 ± 11.60	22.82 ± 20.99	12.79 ± 3.94	12.42 ± 6.66	19.98 ± 17.32
Cr (mg kg^–1^)	4.58 ± 5.53	5.87 ± 3.80	2.03 ± 2.37	4.89 ± 4.31	3.26 ± 4.46	3.93 ± 4.62
Pb (mg kg^–1^)	9.31 ± 5.50	11.90 ± 7.18	6.29 ± 1.98	11.29 ± 5.31	8.00 ± 5.21	11.69 ± 7.36
Cd (mg kg^–1^)	0.50 ± 0.32	0.89 ± 0.42	0.43 ± 0.21	0.98 ± 0.43	0.55 ± 0.28	0.47 ± 0.31
Ni (mg kg^–1^)	3.28 ± 2.54	4.51 ± 2.23	0.43 ± 0.51	4.49 ± 2.53	3.52 ± 2.25	2.58 ± 2.70

a P1—Genésio Dam; P2—Alto da Conceição Bridge; P3—Centro Dam; P4—Santa Luzia Island Canal; P5—East–West Bridge; and P6—Barrocas Dam.

### 2.5 Bioaccumulation and Translocation Factor of Macrophytes

The accumulation of chemical elements in macrophytes depends on several factors, such as physiology and environmental characteristics, and this accumulation is concentrated differently in different parts of the plants (roots, stems, and/or leaves).^35–38^

To determine the bioaccumulation factor of chemical elements in plants (FB), eq 1 proposed by^39^ was used.

1 $$\mathrm{BF}=\frac{C_{\mathrm{aerial}}}{C_{\mathrm{water}}}$$

Where:

BF—Bioaccumulation factor in the aerial part of the plant, dimensionless;

C_aerial_—Concentration of heavy metals and nutrients in the aerial part of the plant, mg kg^–1^;

C_water_—Average concentration of heavy metals and nutrients in river water obtained during the eight campaigns, mg L^–1^.

Translocation is the transfer of chemical elements from the root region to the aerial part of the plant; to estimate this, eq 2 recommended by ref 40 was used.

2 $$\mathrm{TF}=\frac{C_{\mathrm{aerial}}}{C_{\mathrm{root}}}$$

Where:

TF—Translocation factor, dimensionless;

C_aerial_—Concentration of heavy metals and nutrients in the aerial part of the plant, mg kg^–1^;

C_root_—Concentration of heavy metals and nutrients in the plant root, mg kg^–1^.

### 2.6 Statistical Analysis

To identify potential inconsistencies in the data, a preliminary analysis was performed to verify any discrepancies such as identifying missing values, detecting typing errors, duplicates and inconsistencies, verifying units of measurement and standardization, and evaluating outliers. Subsequently, the data was subjected to measures of central tendency (arithmetic mean) and dispersion (standard deviation) for all attributes investigated in the study.

It should be noted that, among the 14 chemical elements evaluated for bioaccumulation (BF) and translocation (TR) factors namely N, P, K, Ca, Mg, Na, Fe, Mn, Cu, Zn, Cr, Pb, Cd, and Ni multivariate statistical analysis was feasible for only eight elements (Zn, Mn, Fe, Cr, Ni, Pb, Cd, and Na). The inclusion of all 14 elements in the phytoremediation data set resulted in a correlation matrix with a determinant close to zero, indicating severe multicollinearity. Consequently, six variables (N, P, K, Ca, Mg, and Cu), which exhibited high correlations with other variables and low variance, were excluded to mitigate multicollinearity effects. Additionally, the preliminary analysis using all 14 elements yielded a Kaiser–Meyer–Olkin (KMO) coefficient below 0.50, which is considered unacceptable. In contrast, the reduced data set comprising eight elements produced a KMO value greater than 0.60, indicating acceptable sampling adequacy.^41^

First, the Pearson correlation matrix (PCM) was constructed to evaluate linear relationships among the variables. In this context, correlation coefficients (r) approaching −1 or +1 indicate strong linear associations, whereas values close to zero indicate little or no correlation. Additionally, the statistical significance of Pearson’s correlation (*p* < 0.05) was applied to assess the bioaccumulation (BF) and translocation (TF) factors of chemical elements in macrophytes, thereby ensuring the reliability of the minimum correlations considered for factor analysis (FA). Furthermore, correlation coefficients with absolute r values greater than 0.50 were classified as strong, those ranging from 0.30 to 0.50 as moderate, and those between 0.00 and 0.30 as weak.^42^

Subsequently, principal component analysis (PCA) was applied to identify the most relevant attributes influencing the BF and TF values of chemical elements in macrophytes across different seasons and sampling locations along the urban stretch of the Apodi–Mossoró River. Accordingly, two vector projection diagrams were constructed for the principal components, representing the attributes evaluated in this study. Kaiser’s criterion (KMO > 0.60) was adopted for this analysis to explain a substantial proportion of the data variability. Based on this criterion, principal component with eigenvalues equal to or greater than 1 and/or those accounting for a cumulative variance exceeding 70% were retained for interpretation.^43,44^

Finally, factor analysis (FA) was applied to facilitate data interpretation and subsequent discussion, as it is based on the correlations among variables. This approach aims to identify and explain variables exhibiting the strongest interrelationships while minimizing the influence of less relevant variables. In this analysis, components with eigenvalues greater than 1 were extracted, and the factor axes were subsequently rotated using the Varimax method to improve interpretability. Factor loadings with absolute values ≥ 0.60 were considered significant. It should be noted that all multivariate statistical analyses were performed using Statistica 14 software.^45^

## 3 Results

### 3.1 Influence of Rainfall and Solar Radiation on Changes in Surface Water Quality

Figure 3 provides the hydroclimatic background needed to interpret the spatial patterns in water chemistry (Table 2) and macrophyte occurrence along the studied urban reach (Figure 1 and 2). In semiarid systems, seasonal rainfall and solar radiation regulate dilution versus evapoconcentration, control particulate mobilization, and influence redox and photochemical conditions that govern nutrient cycling and trace-metal speciation.

In the wetter months (Figure 3), higher rainfall can dilute dissolved constituents but also increases surface runoff and hydraulic disturbance, enhancing the delivery of dissolved/particulate organic matter and urban-derived loads and promoting sediment resuspension. These processes intensify water-sediment exchange and can elevate particle-associated phosphorus as well as redox-sensitive metals. This is consistent with the higher concentrations and variability observed at downstream and urban-proximal sites in Table 2, including increased TKN (up to 11.84 mg L^–1^ at P5) and total P (up to 0.23 mg L^–1^ at P6), together with strong dispersion in Fe and Mn (Fe up to 38.55 mg L^–1^ at P4; Mn up to 17.02 mg L^–1^ at P6). At the molecular scale, these trends are compatible with phosphate release through desorption and/or dissolution of Fe/Mn (oxyhydr)­oxides under reducing microenvironments, as well as metal complexation by natural organic ligands that can stabilize dissolved forms and influence bioavailability to *Eichhornia crassipes*. During drier periods with higher solar radiation (Figure 3), evapoconcentration and more oxidizing/photochemically active conditions can shift speciation and promote readsorption or precipitation, contributing to the contrast between relatively stable major ions and the more site-dependent behavior of nutrients and redox-active elements (Table 2).

Additionally, during the dry season, reduced rainfall combined with higher solar radiation (Figure 3) increases water residence time and promotes evaporative concentration, which raises dissolved-solute levels and ionic strength. Under these conditions, major cations (Na^+^, K^+^, Ca^2+^, and Mg^2+^) tend to become more concentrated, and dissolved nitrogen speciation is influenced by local pH–Eh conditions and microbial transformations (ammonification, nitrification, and denitrification). Elevated irradiance also enhances primary productivity, generating diel oscillations in dissolved oxygen and redox potential that affect the oxidation state and solubility of redox-sensitive elements such as Fe and Mn through precipitation/dissolution of (oxyhydr)­oxide phases and associated sorption of oxyanions (phosphate). In parallel, higher macrophyte metabolic activity increases nutrient uptake and metal accumulation via tissue binding and complexation, contributing to the higher concentrations of nitrogen and selected metals observed at specific sites during the drier months (Table 2).

Furthermore, during the dry season, longer residence times and evaporative concentration increase nutrient availability and can accelerate eutrophication by sustaining larger dissolved N and P pools and strengthening nutrient recycling at the water–sediment interface. In Table 2, total P is similar at P1 to P3 (0.03–0.05 mg L^–1^), whereas the highest mean value occurs at P6 (0.23 mg L^–1^). From an elemental and molecular perspective, higher phosphate under low-flow conditions is consistent with reduced dilution and, locally, greater persistence of dissolved phosphate through desorption from reactive mineral surfaces and/or dissolution of Fe/Mn (oxyhydr)­oxide phases, in addition to association with dissolved organic matter.

Comparison of average tissue water content revealed higher values in *Eichhornia crassipes*, exceeding those observed in *Nymphaea alba* by approximately 6.5–7.5% (Table 3). However, in sites P2 to P6, where *Eichhornia crassipes* was the dominant species, the maximum variation in water content was only 1%. This small variation suggests that tissue water content depended primarily on the species and remained relatively stable despite the marked heterogeneity in river water nutrient levels, anthropogenic pollution inputs, and local geogenic conditions. On the other hand, the high standard deviations associated with fresh and dry biomass indicate that these parameters were more sensitive to environmental variability, reflecting the influence of site-specific pollution sources, geogenic context, and seasonal hydrological changes between dry and rainy periods. Elevated phosphate supports adenosine triphosphate dependent metabolism and the biosynthesis of nucleic acids and phospholipids and favors intracellular orthophosphate and polyphosphate storage, which is consistent with increased growth and tissue hydration. In parallel, higher dissolved nitrogen (Table 2) supplies substrates for amino acid and protein synthesis, further promoting biomass accumulation. Overall, the coincidence of higher P (notably at P6) and greater macrophyte fresh and water masses (P5 and P6) indicates nutrient-enriched conditions during the dry season, with clear implications for eutrophication development.^47^

### 3.2 Bioaccumulation (BF) and Translocation (TF) Factors of Chemical Elements in Macrophytes

Table 6 shows the arithmetic mean values of the bioaccumulation factor (BF) and translocation factor (TF) for chemical elements accumulated in macrophytes at the sampled sites. Overall, BF values greater than 1 for most elements indicate a strong capacity of the macrophytes studied to accumulate nutrients and metals from the aquatic environment. Particularly high BF values were observed for Na, Fe, Mn, Zn, Cr, Pb, Cd, and Ni, highlighting the role of these species as effective sinks for chemical elements present in surface water sources.

**Table 6:** Arithmetic Mean of Bioaccumulation (BF) and Translocation (TF) Factors for Chemical Elements Accumulated in Macrophytes (N = 8)^a^

	P1 (*Nymphaea alba*)		P2 (*Eichhornia crassipes*)		P3 (*Eichhornia crassipes*)		P4 (*Eichhornia crassipes*)		P5 (*Eichhornia crassipes*)		P6 (*Eichhornia crassipes*)	
ChemicalElements	BF	TF	BF	TF	BF	TF	BF	TF	BF	TF	BF	TF
N	18.51	1.41	20.88	1.32	11.57	1.54	7.91	1.92	8.51	1.12	4.44	1.04
P	8.07	1.01	11.59	1.22	15.52	1.07	3.97	1.14	7.67	1.38	5.72	2.03
K	36.76	0.84	45.75	1.34	44.79	0.78	54.19	1.16	2,862.01	1.55	34.46	1.42
Ca	8.77	1.29	0.02	1.67	2.38	1.36	0.01	0.62	0.02	4.24	0.02	1.28
Mg	0.02	1.41	0.04	1.44	11.96	5.98	0.10	0.62	0.04	1.53	0.05	1.80
Na	988.90	1.18	1,215.07	1.12	972.38	0.88	692.18	1.19	1,381.52	1.26	999.10	2.95
Fe	101.26	2.12	3,008.98	1.38	80.57	7.76	31.08	2.65	5,122.39	2.91	2,868.94	1.57
Mn	404.35	1.68	3,370.45	1.44	925.46	9.66	126.58	2.97	1,005.57	1.67	1,718.69	2.62
Cu	2.87	9.32	4.81	2.15	68.57	4.24	5,421.43	13.08	1,504.46	2.72	2,811.25	2.72
Zn	122.73	1.83	34.98	0.51	1694.83	2.37	106.32	1.21	57.20	1.39	34.93	1.48
Cr	7,574.11	1.73	49.88	0.20	11,291.96	3.37	5,448.21	**0.07**	4,906.25	0.25	9,062.50	9.80
Pb	16,669.64	1.04	55.76	1.08	12.86	1.95	20.43	1.15	5.27	0.68	8.31	1.40
Cd	1,245.54	2.79	1,350.89	0.74	774.11	0.71	22.71	0.30	6.38	0.12	1,406.25	2.43
Ni	6,298.21	2.12	6,500.89	1.55	5,344.64	19.86	6,342.86	0.67	5,683.04	0.69	8,806.25	23.23

a P1—Genésio Dam; P2—Alto da Conceição Bridge; P3—Centro Dam; P4—Santa Luzia Island Canal; P5—East–West Bridge; and P6—Barrocas Dam.

The TF values provide insight into the internal mobility of elements within plant tissues. In general, TF values greater than 1 indicate efficient translocation from roots to aerial parts, whereas TF values lower than 1 suggest preferential retention in root tissues. Essential nutrients such as N, P, and K generally exhibited TF values close to or greater than 1, reflecting their physiological demand and high mobility within plant tissues. In contrast, several potentially toxic elements, including Cr, Pb, and Cd, displayed lower TF values at certain sites, indicating a tendency toward root sequestration as a tolerance and detoxification strategy.

### 3.3 Pearson Correlation Matrix of Bioaccumulation (BF) and Translocation (TF) Factors

There was a greater number of significant correlations (*p* < 0.05) between the TF (29%) and the BF index (11%) of the metals present in the biomass of macrophytes (Figure 4).

**Figure 4:**
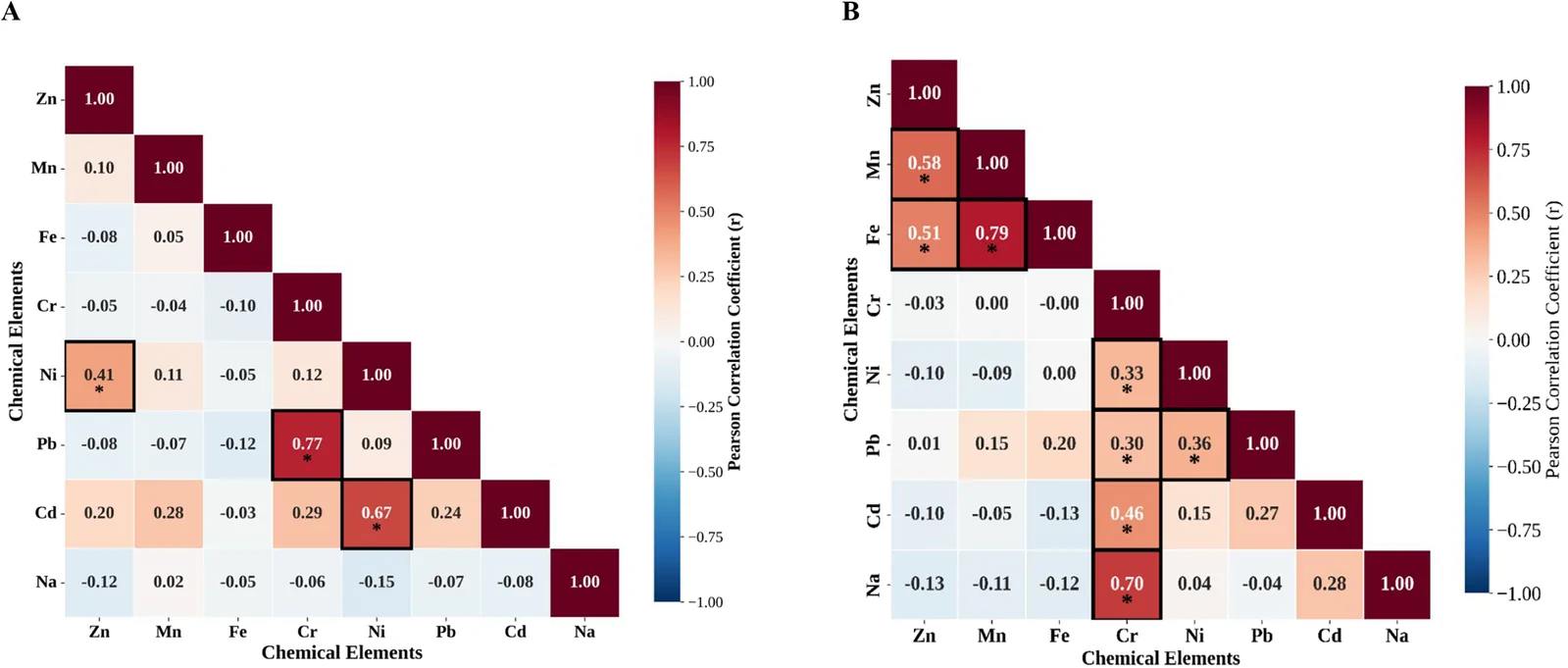
Pearson correlation matrix of bioaccumulation-BF (A) and translocation-TF (B) factors for the chemical elements Zn, Mn, Fe, Cr, Ni, Pb, Cd, and Na. Note: * Indicates statistical significance of Pearson’s correlation (*p* < 0.05).

### 3.4 Principal Component Analysis (PCA) of Bioaccumulation (BF) and Translocation (TF) Factors of Metals Present in the Water of the Apodi-Mossoró River

Principal component analysis (PCA) applied to the BF data for the eight metals (Zn, Mn, Fe, Cr, Ni, Pb, Cd, and Na) revealed four principal components (PCs) with eigenvalues equal to or greater than 1 (Kaiser criterion), which together explained 74.46% of the total variance (Table 7).

**Table 7:** Eigenvalues, Cumulative Variance, and Kaiser Criterion of the Principal Components (PCs) Obtained for the Bioaccumulation Factor (BF) of Metals in Macrophytes

Component	Eigenvalue	Variance (%)	Cumulative Variance (%)	Kaiser
CP1	2.23	27.32	27.32	Yes
CP2	1.62	19.85	47.17	Yes
CP3	1.23	15.01	62.18	Yes
CP4	1.00	12.27	74.46	Yes
CP5	0.81	9.96	84.42	No
CP6	0.57	6.95	91.37	No
CP7	0.53	6.44	97.81	No
CP8	0.18	2.19	100.00	No

Furthermore, the communalities indicated that these four retained PCs adequately represented the original structure of the BF data, revealing a mean of 0.76. All variables showed values greater than 0.69, demonstrating good explanatory power of the model. Fe (0.85) and Cr (0.83) were the best represented elements, classified as excellent, while Zn, Ni, Pb, Cd, and Na showed good representation. Mn showed the lowest communality (0.69), although still adequate, suggesting that part of its variability may be associated with unretained components (Table 8).

**Table 8:** Communalities (h^2^) and Classification of Metal Representativeness in the PCA Model Applied to the Bioaccumulation Factor (BF) in Macrophytes

Metal	h^2^	Classification
Fe	0.85	Excellent
Cr	0.83	Excellent
Zn	0.78	Good
Ni	0.75	Good
Pb	0.74	Good
Cd	0.72	Good
Na	0.71	Good
Mn	0.69	Adequate

In addition, Figure 5 presents biplots 1–2 and 3–4 of BF for the spatiotemporal evaluation of macrophyte phytoremediation of metals present in the water of the Apodi-Mossoró river.

**Figure 5:**
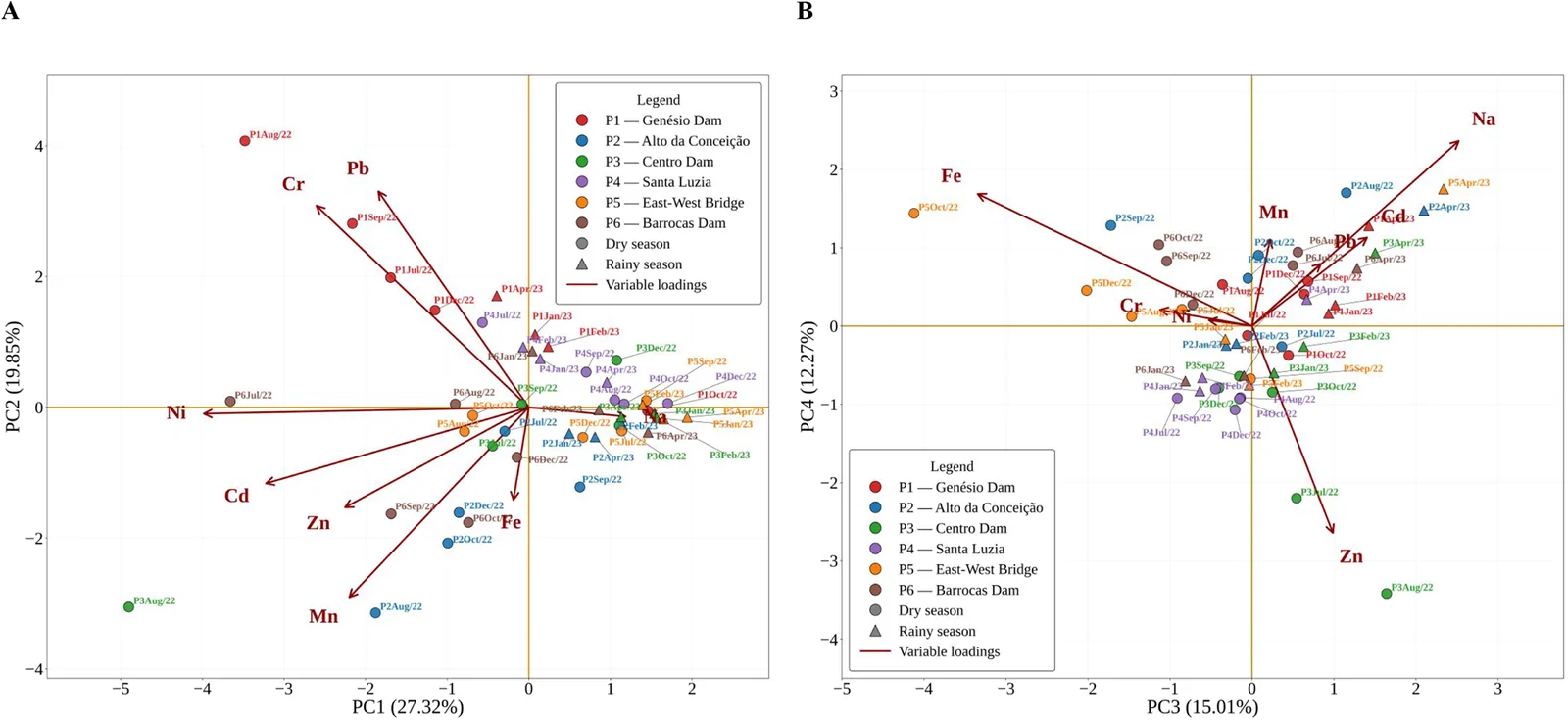
Principal component analysis (PCA) of metal bioaccumulation factors (BF): variable projections and spatiotemporal distribution of BF in macrophytes, highlighting biplots of components 1–2 (A) and 3–4 (B).

Table 9 presents the factor loadings of the correlation type that represents the Pearson correlation between each original variable and its respective principal component, allowing for the direct interpretation of which elements dominate each axis of the PCA of BF.

**Table 9:** Correlation Factor Loadings for Principal Component Analysis (PCA) of the Bioaccumulation Factor (BF)

Metal	CP1	CP2	CP3	CP4
Zn	0,49	–0,33	–0,23	0,61
Mn	0,48	–0,63	–0,05	–0,26
Fe	0,04	–0,31	0,77	–0,39
Cr	0,56	0,67	0,26	–0,05
Ni	0,86	–0,02	0,13	–0,02
Pb	0,40	+0,71	–0,20	–0,19
Cd	0,70	–0,25	–0,33	–0,26
Na	–0,26	–0,03	–0,58	–0,55

Regarding TF, PCA applied to FT data identified three PCs with eigenvalues ≥ 1, explaining 72.18% of the total variance (Table 10).

**Table 10:** Eigenvalues, Cumulative Variance, and Kaiser Criterion of the Principal Components (PCs) Obtained for the Bioaccumulation Factor (TF) of Metals in Macrophytes

Component	Eigenvalue	Variance (%)	Cumulative Variance (%)	Kaiser
CP1	2.47	30.27	30.27	Yes
CP2	2.17	26.61	56.88	Yes
CP3	1.25	15.30	72.18	Yes
CP4	0.79	9.69	81.87	No
CP5	0.60	7.34	89.21	No
CP6	0.49	5.99	95.21	No
CP7	0.20	2.48	97.68	No
CP8	0.19	2.32	100.00	No

The mean communality of TF was 0.74 (Table 11). It is noteworthy that Cr, Mn, Na, and Fe were excellently represented by the three retained components. Meanwhile, Cd showed the lowest communality (0.46), indicating that a substantial portion of its variability in translocation is not captured by the retained components and may be related to specific sampling point factors not fully represented in the PCA model.

**Table 11:** Communalities (h^2^) and Classification of Metal Representativeness in the PCA Model Applied to the Bioaccumulation Factor (TF) in Macrophytes

Metal	h^2^	Classification
Cr	0.88	Excellent
Mn	0.86	Excellent
Na	0.86	Excellent
Fe	0.82	Excellent
Pb	0.72	Good
Ni	0.64	Adequate
Zn	0.63	Adequate
Cd	0.46	Low

Figure 6 shows biplots 1–2 and 3–4 of TF for the spatiotemporal evaluation of phytoremediation of metals present in the water of the Apodi-Mossoró river by macrophytes.

**Figure 6:**
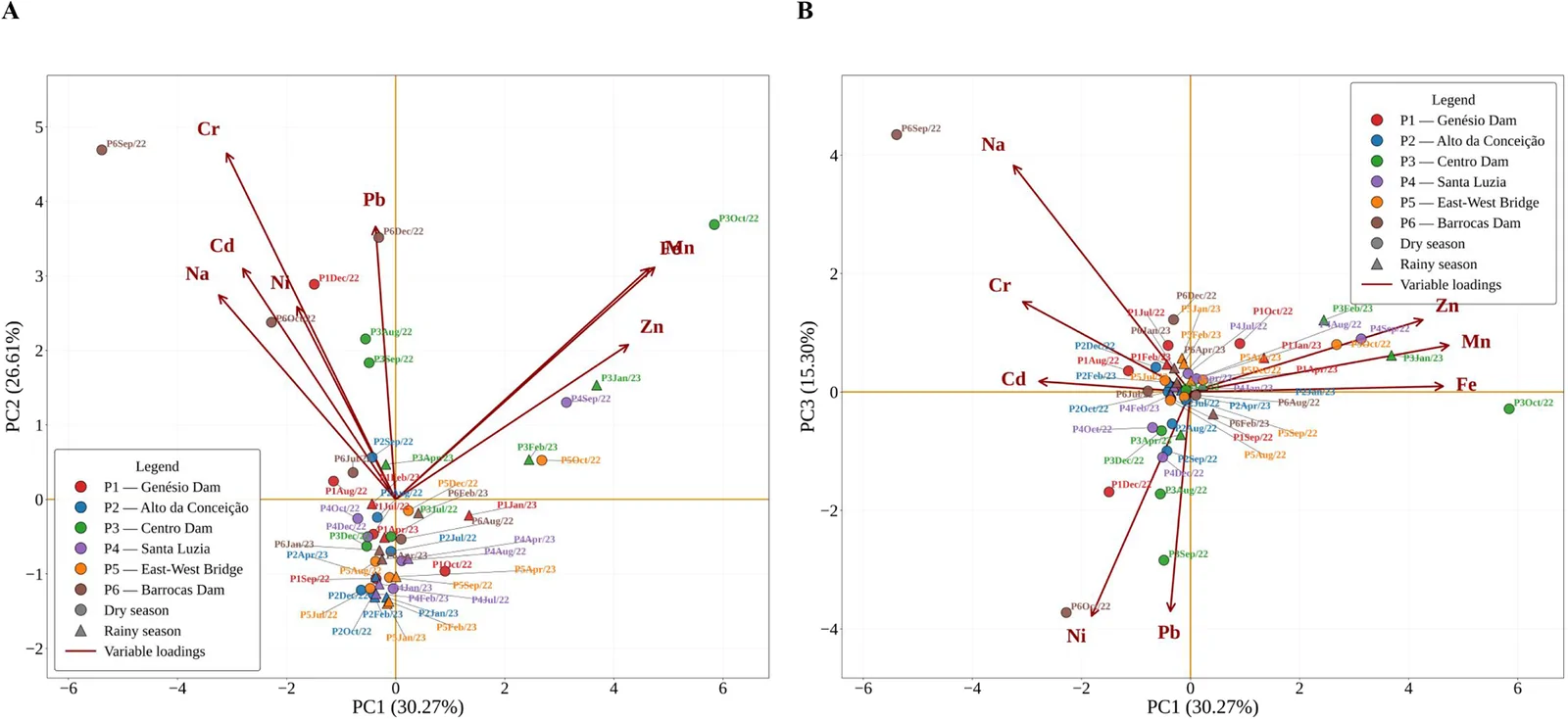
Principal component analysis (PCA) of metal bioaccumulation factors (TF): variable projections and spatiotemporal distribution of TF in macrophytes, highlighting biplots of components 1–2 (A) and 3–4 (B).

Table 12 shows the correlation type factor loadings, which correspond to the Pearson correlations between each original variable and its associated principal component. This approach enables a straightforward identification of the elements that most strongly contribute to each axis of the TF/PCA.

**Table 12:** Correlation Factor Loadings for Principal Component Analysis (PCA) of the Bioaccumulation Factor (TF)

Metal	CP1	CP2	CP3
Zn	0,69	0,34	0,20
Mn	0,77	0,50	0,13
Fe	0,75	0,50	0,02
Cr	–0,50	0,75	0,25
Ni	–0,30	0,42	–0,62
Pb	–0,06	0,60	–0,60
Cd	–0,46	0,50	0,03
Na	–0,53	0,45	0,62

### 3.5 Factor Analysis (FA) for Bioaccumulation (BF) and Translocation (TF) Factors

As a complement to the PCAs, Table 13 presents the results of the factor analyses, highlighting the retention of four factors for BF and three factors for TF, along with their respective eigenvalues, percentages of variance explained, and cumulative variance.

**Table 13:** Factor Loadings Derived from Macrophyte Bioaccumulation (BF) and Translocation (TF) Variables, along with Their Corresponding Eigenvalues, Total Variance, and Cumulative Total Variance^a^

BF					TF			
Attributes	F1	F2	F3	F4	Attributes	F1	F2	F3
Zn	**0.66**	–0.25	0.22	0.24	Zn	**0.78**	–0.05	–0.11
Mn	0.37	–0.07	**–0.61**	–0.35	Mn	**0.92**	–0.02	0.03
Fe	–0.10	–0.10	0.07	**–0.88**	Fe	**0.89**	–0.07	0.12
Cr	0.08	**0.92**	0.03	0.05	Cr	0.04	**0.89**	0.27
Ni	**0.87**	0.09	0.05	0.03	Ni	–0.12	0.11	**0.78**
Pb	0.03	**0.92**	0.06	0.07	Pb	0.17	0.10	**0.82**
Cd	**0.77**	0.33	–0.23	–0.12	Cd	–0.10	0.59	0.32
Na	–0.26	–0.06	**–0.76**	0.30	Na	–0.08	**0.90**	–0.19
EV	2.22	1.68	1.06	1.03	EV	2.42	2.13	1.22
TV (%)	27.32	19.85	15.01	12.27	TV (%)	30.27	26.61	15.30
CTV (%)	27.32	47.17	62.18	74.45	CTV (%)	30.27	56.88	72.18

a For interpretation purposes, factor loadings ≥ 0.60 were considered significant and are highlighted in bold. F—Factor; EV—Eigenvalues; TV—Total variance; CTV—Cumulative total variance.

## 4 Discussion

The high standard deviation values for some characteristics in Tables 2, 3, 4, and 5 indicate strong environmental heterogeneity, which is consistent with the hydrochemical dynamics of semiarid Brazilian rivers subject to seasonal hydrological variability, evapoconcentration, geogenic influence, and localized sources of anthropogenic pollution. In the case of the Apodi-Mossoró River, the lack of universal basic sanitation coverage may contribute to point and diffuse pollution sources along its course, including the discharge of domestic sewage, the disposal of urban solid waste, drainage inputs, and other types of pollution. Therefore, some sampling points showed considerably higher concentrations than others, increasing the standard deviation.

The results show pronounced differences in BF and TF between *Nymphaea alba* and *Eichhornia crassipes* (Table 6). These contrasts reflect species-specific morphology and physiology that govern elemental sorption at root surfaces, membrane transport, intracellular complexation, and tissue-level partitioning, as well as spatial controls associated with anthropogenic and/or geogenic sources along the reach of the water body (Figure 2). In addition, climatological variables such as rainfall and global solar radiation, influence dilution/evapoconcentration, water-sediment exchange, and redox conditions that shift aqueous speciation and bioavailability of nutrients and trace metals, thereby influencing BF and TF patterns.

*Nymphaea alba* exhibited its highest BF and TF values for Pb and Cu, whereas the lowest values were observed for Mg and K. However, *Eichhornia crassipes* showed higher BF and TF values for Cr and Ni, while comparatively lower values were observed for Ca and Cr. Notably, higher population densities of *Nymphaea alba* observed during the dry season may partly explain the elevated BF values for Pb, likely due to increased biomass availability and prolonged interaction with contaminated water. Similarly, *Eichhornia crassipes* is widely recognized for its effectiveness in extracting a broad range of chemical elements from aquatic environments, which makes it a frequently employed species in phytoremediation strategies. This macrophyte exhibits rapid growth and high biomass productivity and tolerates elevated loads of macro and micronutrients as well as potentially toxic trace metals. At the elemental and molecular levels, its accumulation capacity is supported by metal binding at root and cell-wall functional groups, membrane transport into tissues, and intracellular chelation and sequestration (via thiol- and carboxyl-containing ligands), enabling the uptake and storage of elements such as Cd, Pb, Cr, Zn, Sn, and Ag.^14^

A macrophyte is considered to have phytoremediation potential when BF > 1 ^12^; under this criterion, 86% of the evaluated elements (N, P, K, Na, Fe, Mn, Cu, Zn, Cr, Pb, Cd, and Ni) showed BF > 1, while only Mg and Ca exhibited BF < 1 in some of the points, with Mg below unity in P1, P2, P4, P5, and P6, and Ca in P2, P4, P5, and P6 (Table 6). It is noteworthy that the highest BF for the macronutrient N was observed in P2 (20.88), while the lowest occurred in P6 (4.44). For P, the highest BF was recorded in P3 (15.52) and the lowest in P4 (3.97), corroborating the association between environments with high concentrations of N and P and eutrophic conditions (Table 2). Among the essential macronutrients, K stood out for presenting the highest BF (bioaccumulation) at all points, with extremely high accumulation at P5 (2862.01), contrasting with lower values at other locations (34.46–54.19), corroborating with BF values >1 found by^46^ in the Apodi-Mossoró river. For Ca and Mg, the highest BF were recorded at P1 (8.77) and P3 (11.96), respectively, while the lowest values occurred predominantly at points dominated by *Eichhornia crassipes*. Na showed high bioaccumulation at all points, ranging from 692.18 (P4) to 1381.52 (P5), evidencing high tolerance of macrophytes to this element.

The micronutrients Fe and Mn showed high spatial variability. Fe exhibited the lowest BF values at P4 (31.08) and P3 (80.57), while extremely high values were observed at P5 (5,122.39) and P2 (3,008.98). For Mn, the highest BF occurred at P2 (3,370.45), followed by P6 (1,718.69), while the lowest was recorded at P4 (126.58). BF values >1 for Fe and Mn corroborate the results of^46^ in the Apodi-Mossoró river. Cu showed low BF values at P1 (2.87) and P2 (4.81), but extremely high bioaccumulation at P4 (5,421.43) and P6 (2,811.25), even with trace concentrations in the water (Table 2), indicating high absorption efficiency by macrophytes. For Zn, the lowest bioaccumulation factor (BF) was observed at P6 (34.93) and the highest at P3 (1,694.83).

Regarding metals, Cr showed very high BF values at most points, particularly at P3 (11,291.96), P6 (9,062.50), and P1 (7,574.11), despite occurring mostly at trace levels in the aquatic environment. In contrast, P2 showed reduced BF (49.88), even with a higher concentration of Cr in the water, indicating lower assimilation efficiency at this point. It is emphasized that another adaptation of plants that remove Cr refers to the ability to immobilize Cr in the cell wall, preventing its movement to areas such as mitochondria and chloroplasts.^47^ Ni showed high bioaccumulation at all sampled locations, with values ranging from 5,344.64 (P3) to 8,806.25 (P6), demonstrating a high affinity of macrophytes for this element when available. Thus, depending on the availability of the chemical element in the environment, plants have a great capacity to accumulate Ni in their biomass.^48,49^

Among the highly toxic metals, Pb showed bioaccumulation values lower than 56 in P2–P6, except in P1, where an extremely high value (16,669.64) was observed, possibly associated with local anthropogenic activities (vehicle washing, water collection in trucks, and proximity to highways) and the macrophyte’s affinity for absorbing this metal. Pb was prominent in bioaccumulation by the macrophyte *Nymphaea alba*. In the study by,^50^ the order of bioaccumulation was Pb > Cd > Zn. Pb accumulates differently in different parts of plant tissue, especially in the roots.^51^ Thus, Pb levels in plants tend to decrease in the following order: roots, leaves, stem, inflorescence, seeds.^52^ Some Pb hyperaccumulator plants have anatomical and physiological characteristics that inhibit its transport within the plant, creating barriers that hinder the transport of Pb to other parts.^53^ Cd bioaccumulation was more pronounced in P1 (1,245.54), P2 (1,350.89), and P6 (1,406.25), while lower values were recorded in P4 and P5. Tests using Cd and Zn with the macrophyte *Eichhornia crassipes* showed a high capacity for bioaccumulation of these elements, mainly in the root region, compared to the aerial part.^54^ For the macrophytes *Nymphaea alba* (P1) and *Eichhornia crassipes* (P2 to P6), there was distinct behavior in terms of greater and lesser bioaccumulation capacity. In *Nymphaea alba*, Pb was the most bioaccumulated chemical element, while Mg was the least bioaccumulated. However, *Eichhornia crassipes* showed alternation between the most bioaccumulated chemical elements (Ni and Cr). In P2, P4, and P5, Ni was more bioaccumulated, and in P3 and P6, Cr showed the highest FB values. Meanwhile, the least bioaccumulated chemical element by the macrophyte *Eichhornia crassipes* was Ca in P2 to P6.

As shown in Table 6, both macrophytes generally exhibited TF ≥ 1 for most elements, indicating efficient internal translocation after root uptake. Following adsorption at root surfaces and entry into root cells, elements are redistributed via xylem transport-either stored through intracellular complexation/sequestration or delivered to shoots and leaves depending on their chemical form and binding affinity. *Eichhornia crassipes* displayed a broader TF range and, overall, higher TF values than *Nymphaea alba*, indicating greater variability and a stronger propensity for shootward partitioning of the accumulated elements. Ref 55 report that this transport capacity depends on specific physiological characteristics of the species, including antioxidant defense mechanisms and the compartmentalization of elements within vesicular structures. Furthermore, the translocation of chemical elements is controlled by root pressure and transpiration-induced flow associated with foliar respiration.^56^

Regarding the macronutrients N, P, and K, elements of great importance for plants, N and P were concentrated in the aerial part at all sampling points (P1 to P6). However, the concentration of K was lower at P1 (0.84) and P3 (0.78), suggesting that this element remained more concentrated in the root region. It is noteworthy that K and P are considered limiting elements for the development of several plants in Brazil,^57^ and are also present in urban sewage. Probably these lower inputs of N and P in the water source resulted in lower biomass in plants at points P1 and P3 compared to the others (Table 3). Ca and Mg showed TF > 1 at almost all points, except at P4, where both elements showed TF of 0.62, with these chemical elements concentrated in the roots. Na was not translocated to the aerial part in all samples analyzed in the plant tissue, except at P3 (0.88).

The micronutrients Fe, Mn, and Cu showed TF > 1, indicating that these elements were absorbed by the roots and transported to the aerial part of the macrophytes. Among those mentioned, it is noteworthy that the greatest translocations occurred in Mn and Cu. In this sense, the work of^58^ revealed high concentrations of Mn and Cu in the plant tissue of *Pennisetum purpureum*. Similarly, Zn and Pb showed TF > 1 in five points, except for P2 (0.51) for Zn and P5 (0.68) for Pb, revealing a concentration of these metals in the root system (Table 5). Thus,^59^ also identified TF < 1 for Pb in three of the treatments studied, evidencing accumulation of this metal in the root system. The TF values of Cr were elevated in half of the points, while the other points showed TF < 1. A study conducted by^60^ found that high Cr levels caused a reduction in the protein content of *Nymphaea alba*. Similarly, at points P3 (0.71), P4 (0.30), and P5 (0.12), Cd also showed TF < 1. Ni, in most cases, showed translocation to the aerial part of the plant (Table 4), except for P4, which recorded 0.67, and P6, which recorded 0.69. This element showed the highest TF value (23.23) at P5.

In the analysis of the arithmetic mean values of the bioaccumulation factor (FB) and translocation factor (TF) in macrophytes (Figure 4), correlation coefficients with absolute r values greater than 0.50 were classified as strong, those between 0.30 and 0.50 as moderate, and those less than 0.30 as weak.^42^ Based on this classification, the BF correlation matrix revealed two strong positive associations between Cr and Pb (r = 0.77) and between Ni and Cd (r = 0.67), indicating a high degree of linear dependence. A moderate correlation was observed between Zn and Ni (r = 0.41). The TF correlation matrix revealed strong positive correlations between Fe–Mn (r = 0.79), Zn–Mn (r = 0.58), Zn–Fe (r = 0.51), and Cr–Na (r = 0.70), indicating a high degree of linear association. Moderate correlations were observed between Cr–Cd (r = 0.46), Cr–Ni (r = 0.33), Ni–Pb (r = 0.36) and Cr–Pb (r = 0.30).

Figure 5 shows that the PCA of FB revealed four CPs divided into two biplots. In biplots 1–2, CP1 and CP2 were integrated, totaling 47.17% of the variance. Specifically, CP1 reflects the overall capacity for metal bioaccumulation by macrophytes, where the relative importance of each variable in defining this component was Ni (33.06%) > Cd (21.71%) > Cr (14.15%) > Zn (10.69%) > Mn (10.13%) > Pb (7.12%) > Na (3.06%) > Fe (0.08%). Thus, Ni (r = 0.86), Cd (r = 0.70), and Cr (r = 0.56) showed the strongest positive charges (Table 9), indicating that CP1 mainly captures the combined bioaccumulation of these three toxic metals. On the other hand, the moderate positive contributions of Zn (0.49), Mn (0.48), and Pb (0.40) suggest an overall accumulation gradient, where higher scores in CP1 correspond to locations and periods of high overall metal bioaccumulation. Na showed a weak negative charge (−0.26), suggesting a slightly antagonistic relationship with the accumulation of toxic metals in plant tissue. The dominance of Ni in this component is consistent with the high FB values observed at all sampling points (FB > 5,000), reflecting the ability of both *Eichhornia crassipes* and *Nymphaea alba* to accumulate this element even from very low concentrations in water.

CP2, on the other hand, distinguishes two distinct bioaccumulation patterns, with Pb (0.71) and Cr (0.67) showing strong positive charges, while Mn (−0.63) exhibited a strong negative charge (Table 9). This opposition indicates that CP2 captures a contrast between the bioaccumulation of Pb and Cr, which tend to co-occur, versus Mn, which follows a distinct absorption pattern. The strong positive correlation between Cr and Pb bioaccumulation (r = 0.77 in Figure 4) supports this interpretation, suggesting shared sources or similar accumulation mechanisms for these two toxic metals in macrophyte tissue. The clustering of P1 samples in the positive quadrant of CP2 is consistent with the very high Pb bioaccumulation observed at the Genésio Dam (FB = 16,669.64), likely influenced by the proximity of the BR-304 highway. However, in CP2 the relative importance of each variable in defining this component was Pb (31.44%) > Cr (27.39%) > Mn (24.35%) > Zn (6.80%) > Fe (6.03%) > Cd (3.90%) > Na (0.06%) > Ni (0.03%).

In the second biplot of BF, which explained 27.28% of the total variance (Figure 5), CP3 stood out due to the opposition between Fe (0.77) and Na (−0.58), indicating that this component likely represents distinct geochemical dynamics associated with these two elements (Table 9). Fe bioaccumulation was more related to its availability in the water, possibly influenced by processes such as bank erosion during the rainy season. On the other hand, Na accumulation reflected the salinity condition in the soil-water system, in which macrophytes demonstrated behavior compatible with halophytic strategies. Thus, the opposing directions of these elements in the biplot suggest that the environmental conditions that favor Fe bioaccumulation do not necessarily coincide with those that intensify Na absorption, especially during periods of drought, when there is a higher concentration of salts in the aquatic environment. In CP3, the relative importance of each variable in defining this component was Fe (48.70%) > Na (27.74%) > Cd (8.77%) > Cr (5.73%) > Zn (4.27%) > Pb (3.22%) > Ni (1.35%) > Mn (0.22%).

CP4 mainly separates Zn (0.61) from Na (−0.55), with a moderate negative contribution from Fe (−0.39) presented in Table 9. This may reflect local contamination patterns, since Zn showed highly variable FB values between points (from 34.93 at P6 to 1,694.83 at P3), contrasting with the more uniform bioaccumulation of Na. It is noteworthy that in CP4 the relative importance of each variable in defining this component was: Zn (37.45%) > Na (29.81%) > Fe (15.14%) > Cd (6.96%) > Mn (6.83%) > Pb (3.53%) > Cr (0.24%) > Ni (0.03%).

Regarding the PCA of TF, the first biplot showed a cumulative variance of 56.88%, while the second explained 45.57% (Figure 6). In PC1, the relative importance of the variables in defining this component followed the order: Mn (24.01%) > Fe (23.01%) > Zn (19.47%) > Na (11.25%) > Cr (10.21%) > Cd (8.39%) > Ni (3.52%) > Pb (0.15%). This component mainly represents the translocation of essential micronutrients, since Mn (0.77), Fe (0.75), and Zn (0.69) showed the highest positive charges (Table 12). This pattern indicates that PC1 reflects the coordinated movement of these elements from the roots to the aerial part of macrophytes. Conversely, Na (−0.53) and Cr (−0.50) exhibited negative charges, suggesting that the greater translocation of essential micronutrients may be associated with the lower mobility of these elements in the plant. From a physiological point of view, this behavior is consistent, since Mn, Fe, and Zn play fundamental roles in photosynthesis, enzymatic activation, and carbohydrate metabolism, and are therefore actively directed to leaf tissues. Furthermore, the strong correlation between Fe and Mn translocation (r = 0.79), observed in the Figure 4, reinforces this pattern of joint transport, possibly related to shared mechanisms of mobilization and transport via the xylem.

Thus, the relative importance of the variables in defining CP2 followed the order: Cr (26.08%) > Pb (16.31%) > Mn (11.74%) > Cd (11.67%) > Fe (11.65%) > Na (9.14%) > Ni (8.17%) > Zn (5.24%). This component reflects the intensity of translocation for all chemical elements. Where Cr (0.75) showed the strongest charge (Table 12), followed by Pb (0.60), Mn (0.50), Cd (0.50), and Fe (0.50). All charges in CP2 were positive, indicating that this component captures a translocation gradient. Furthermore, higher scores in CP2 correspond to samples where multiple elements were translocated simultaneously at high rates, regardless of whether they are essential or toxic. The strong correlation between the translocation of Na and Cr (r = 0.70) supports the cotransport of these elements in this component.

In CP3 there was opposition between Na (0.62) and the pair Ni (−0.62)/Pb (−0.60) as described in Table 12, this component captures a contrast between the translocation of Na to the aerial parts and the retention of Ni and Pb in the root system. This pattern is consistent with the exclusion mechanism described for toxic metals, where plants create barriers to limit the transport of toxic elements such as Pb and Ni to photosynthetically active tissues. The high FT values for Ni in P3 (19.86) and P6 (23.23) represent notable exceptions to this general pattern. Furthermore, the relative importance of the variables in defining this component was: Na (30.95%) > Ni (30.36%) > Pb (29.17%) > Cr (4.96%) > Zn (3.15%) > Mn (1.32%) > Cd (0.07%) > Fe (0.02%).

PCA reveals fundamental differences between bioaccumulation and translocation processes. While bioaccumulation is driven by the absorption of toxic metals (Ni, Cd, and Cr), intensifying during the dry season when pollutant concentrations increase, translocation is dominated by the active transport of essential micronutrients (Mn, Fe, and Zn), being more pronounced during the rainy season when plant metabolic activity increases. Thus, macrophytes in the Apodi-Mossoró River function both as passive accumulators of environmental contaminants and as active transporters of essential elements, supporting their dual role in phytoremediation and nutrient cycling in the ecosystem.

The FA applied to the BF and TF data sets revealed distinct multivariate structures influencing chemical element behavior in macrophytes (Table 7). For FA/BF, four factors with eigenvalues greater than 1 were retained, explaining 74.45% of the total variance. Factor 1 (27.32%) was primarily associated with Ni, Cd and Zn. Factor 2 (19.85%) was dominated by Cr and Pb. Factor 3 (15.01%) showed strong loadings for Na and Mn, while Factor 4 (12.27%) was mainly influenced by Fe. For FA/TF, three factors were extracted, cumulatively accounting for 72.18% of the total variance. Factor 1 (30.27%) was strongly associated with Mn, Fe and Zn indicating coordinated translocation of essential micronutrients. Factor 2 (26.61%) showed high loadings for Cr and Na. Factor 3 (15.30%) was dominated by Pb and Ni, reflecting restricted or selective translocation mechanisms typically associated with potentially toxic elements.

The results obtained for the BF and TF indices corroborate the hypotheses raised in this study. Initially, marked differences were evidenced between *Nymphaea alba* and *Eichhornia crassipes* regarding the capacity for absorption of chemical elements and intraplant partitioning, indicating distinct accumulation and translocation strategies under semiarid conditions. Additionally, it was found that *Eichhornia crassipes* presented greater phytoremediation potential, especially in the stretches of the water body most affected by anthropogenic pollution. Furthermore, the high spatial and temporal variability of water quality indicators, influenced by local climatic characteristics, with rainfall concentrated in a short period and a prolonged dry season, reinforces the importance of these interspecific differences in the response of macrophytes to contamination gradients in the aquatic environment.^61^

## 5 Conclusion

The phytoremediation efficiency for nutrients and trace metals in surface waters of the Brazilian semiarid region is highly variable and depends on macrophyte species, climatic seasonality, anthropogenic inputs, and local geogenic controls. Together, these drivers regulate elemental speciation and phase partitioning (dissolved, colloidal, and particulate), including oxidation state, aqueous complexation with inorganic ions and natural organic ligands, and sorption onto mineral and organic surfaces. In turn, these molecular-scale processes determine bioavailability and govern plant uptake mechanisms (surface adsorption, membrane transport, and chelation) and subsequent internal redistribution among roots, stems, and leaves via ligand binding and sequestration in cellular compartments.

*Nymphaea alba* and *Eichhornia crassipes* have proven to be robust bioindicators of anthropogenic contamination by nutrients and metals, consistently responding to spatial and environmental variability, while exhibiting distinct physiological strategies for bioaccumulation and translocation.

*Nymphaea alba* showed predominantly accumulating behavior, with hyperaccumulation potential for Pb, Cr, Ni, Cd, and Na, indicating a high affinity for these elements and highlighting its suitability as an indicator of persistent metal pollution in urban aquatic environments.

*Eichhornia crassipes* stood out for its high physiological plasticity, alternating between root exclusion mechanisms (particularly for Ca and Mg at specific points) and hyperaccumulation of K, Na, Fe, Mn, Cu, Cr, and Ni, confirming its high efficiency in phytoremediation and suitability for remediation and management strategies in contaminated semiarid aquatic systems.

The appropriate selection of macrophyte species, combined with a detailed understanding of local environmental conditions, is essential to maximize the efficiency of phytoremediation and support the use of aquatic macrophytes as effective tools for environmental monitoring in semiarid regions.
